# Walking parameters of older adults from a lower back inertial measurement unit, a 6-year longitudinal observational study

**DOI:** 10.3389/fnagi.2022.789220

**Published:** 2022-09-08

**Authors:** Morad Elshehabi, Silvia Del Din, Markus A. Hobert, Elke Warmerdam, Ulrike Sünkel, Tanja Schmitz-Hübsch, Lisa Marie Behncke, Sebastian Heinzel, Kathrin Brockmann, Florian G. Metzger, Christian Schlenstedt, Lynn Rochester, Clint Hansen, Daniela Berg, Walter Maetzler

**Affiliations:** ^1^Department of Neurology, Christian-Albrechts-University, Kiel, Germany; ^2^Translational and Clinical Research Institute, Faculty of Medical Sciences, Newcastle University, Newcastle upon Tyne, UK; ^3^German Center for Neurodegenerative Diseases DZNE, Tübingen, Germany; ^4^Center for Neurology and Hertie Institute for Clinical Brain Research, Department of Neurodegeneration, University of Tübingen, Tübingen, Germany; ^5^Experimental and Clinical Research Center, a cooperation of Max-Delbrueck Center for Molecular Medicine and Charité - Universitätsmedizin Berlin, Berlin, Germany; ^6^Department of Psychiatry and Psychotherapy, University Hospital Tübingen, Tübingen, Germany; ^7^Geriatric Center, University of Tübingen, Tübingen, Germany; ^8^Newcastle Upon Tyne Hospitals NHS Foundation Trust, Newcastle upon Tyne, UK

**Keywords:** gait, older adults, normative data, inertial measurement unit (IMU), aging

## Abstract

Gait changes during aging and differs between sexes. Inertial measurement units (IMUs) enable accurate quantitative evaluations of gait in ambulatory environments and in large populations. This study aims to provide IMU-based gait parameters’ values derived from a large longitudinal cohort study in older adults. We measured gait parameters, such as velocity, step length, time, variability, and asymmetry, from straight, self-paced 20-m walks in older adults (four visits: 715/1102/1017/957 participants) every second year over 6 years using an IMU at the lower back. Moreover, we calculated the associations of gait parameters with sex and age. Women showed lower gait speed, step length, step time, stride time, swing time, and stance time, compared to men. Longitudinal analyses suggest that these parameters are at least partly deteriorating within the assessment period of 2 years, especially in men and at an older age. Variability and asymmetry parameters show a less clear sex- and age-associated pattern. Altogether, our large longitudinal dataset provides the first sex-specific information on which parameters are particularly promising for the detection of age-related gait changes that can be extracted from an IMU on the lower back. This information may be helpful for future observational and treatment studies investigating sex and age-related effects on gait, as well as for studies investigating age-related diseases.

## Introduction

Gait, a highly complex movement that is relevant for daily living, changes with age (DeVita and Hortobagyi, 2000; McGibbon and Krebs, 2001; Ko et al., 2012). Parameters that are particularly prone to age-related changes are gait speed, step and stride length, and variability (DeVita and Hortobagyi, 2000; Brach et al., 2010). Gait is also different between women and men (Kerrigan et al., 1988a; Lee and Grimson, 2002; Bohannon and Williams Andrews, 2011; Ko et al., 2011; Makihara et al., 2011). For example, women have shorter duration and length of steps and higher gait symmetry in terms of limb acceleration (Auvinet et al., 2002).

Gait changes also occur in age-related diseases. Gait parameters can serve as biomarkers to predict and diagnose these diseases and observe their progress (Verghese et al., 2002; Postuma et al., 2012). Specifically, gait parameters associated with the presence of, and progression in, Parkinson’s disease include gait speed, step length, and gait variability (Morris et al., 1998; Lord et al., 2011). Another example is Alzheimer’s disease, which is associated with slower gait speed, difficulties maintaining balance, and clearing obstacles (Alexander et al., 1995). To define the deviation from “normal” of these parameters as early as possible, reference data are still necessary.

Gait parameters can be categorized into spatial and temporal domains (Zijlstra and Hof, 2003). According to a study by Lord et al. (2013), gait can also be classified into relatively independent domains, with pace, rhythm, variability, symmetry, and postural control being probably the most important ones. These domains may be differently affected by sex, age, and disease (Sudarsky, 1990; Shimada, 1996; Callisaya et al., 2009) and can be assessed even with a single inertial measurement unit (IMU), e.g., on the lower back (Del Din et al., 2016). In fact, IMUs can provide a new dimension of granularity for gait analysis and are increasingly used in research studies (Horak et al., 2015; Maetzler and Rochester, 2015). IMUs are microelectromechanical systems which can measure multiple degrees of freedom (e.g., 3D accelerometers, 3D gyroscopes, and 3D magnetometers). They are lightweight, economic, and unobtrusive. These features make them ideal tools for measuring large numbers of participants and for longitudinal observations (Tao et al., 2012). This is a relevant aspect, as most of the reference values we have to date for gait parameters were in small, mostly cross-sectional cohorts, which were assessed in even more artificial environments (Oberg et al., 1993; Auvinet et al., 2002; Ko et al., 2010; Bohannon and Williams Andrews, 2011).

In this study, we report gait parameters extracted from an IMU worn at the lower back and analyzed with a validated algorithm, in a large cohort of older adults, to contribute to the development of normative values for gait in the elderly. This study includes four visits within an observation period of 6 years.

## Methods

### Cohort

This longitudinal observational study includes data from 715 (first visit)/1,102 (second visit, 455 new participants)/1,017 (third visit, 31 new participants) and 957 (fourth visit) older adults. Participants were assessed every second year using an IMU worn at the lower back. We excluded a total of 40 participants who did not perform the assessments included in this analysis, and hence, they had no IMU data. An overview of the cohort size in each visit is presented in Table 1.

**Table 1:** An overview of the number of participants in the first four visits of the TREND study.

**Parameter**	**Visit 1**	**Visit 2**	**Visit 3**	**Visit 4**	**Total**	**Dropouts**
BL	715	0	0	0	**715**	
FU1	455	647	0	0	**1,102**	**68**
FU2	31	399	587	0	**1,017**	**124**
FU3	0	47	375	340	**957**	**195**
**Total**	**1,201**	**1,093**	**962**	**340**		**387**

BL, Baseline; FU, Follow-Up.

Participants took part in the *Tuebinger Evaluation of Risk factors for Early detection of Neurodegenerative Disorders* (TREND) study^1^ (Gaenslen et al., 2014), which has been performed at the Neurology Department of the University Hospital Tübingen, Germany. The study was approved by the ethics committee of the medical faculty of Eberhard Karls University of Tübingen and the University Hospitals of Tübingen (Nr. 90/2009BO2) and was performed in accordance with the Declaration of Helsinki. All participants provided written informed consent. Inclusion criteria were being at least 50 years old, and free of relevant movement disorders as defined by clinical investigation. Exclusion criteria were any significant visual or hearing impairment, diagnosis of a neurodegenerative disease, inflammatory central nervous system diseases or stroke, and the administration of antipsychotic or dopaminergic drugs. Participants underwent neurological examination including the motor part of the Movement Disorder Society-sponsored revision of the Unified Parkinson’s Disease Rating Scale (MDS-UPDRSIII; Goetz et al., 2008) by MDS approved clinicians. The following clinical tests and questionnaires were performed: Mini-Mental State Examination (MMSE), Trail Making Test (TMT) A and B, Beck’s Depression Inventory (BDI)-I and the International Standard Classification of Education (ISCED; Reitan, 1958; Folstein et al., 1975; UNESCO Institute of Statistics, 2011). Moreover, participants provided personal, social, medical, falls, mood, and family history, including history of skeleton muscular diseases or muscle stiffness as well as grip force measurement (average of two attempts from each hand; Hausdorff et al., 1997), medication plan, smoking habits, and alcohol consumption (Callisaya et al., 2009). Table 2 provides an overview of demographic and clinical data. Study data have been collected and managed using REDCap electronic data capture tools hosted at the University of Tübingen (Harris et al., 2009). The study protocol is described in detail in Gaenslen et al. (2014).

**Table 2:** Demographic and clinical data.

**Parameter**	**Women**	**Men**
N (%)	589 (50.7)	572 (49.3)
Age (years)	63.2 (6.9)	63.9 (6.9)
Height (m)	1.65 (0.1)	1.77 (0.1)
Weight (kg)	69.5 (13.2)	82.4 (12.1)
BMI (kg/m2)	25.7 (4.8)	26.4 (3.6)
ISCED (0–6)	3	5
MDS-UPDRS III (0–132)	1.1 (2.7)	1.4 (2.5)
Number of medications	2.1 (1.9)	1.8 (2.1)
Fallers (%)	15.2	7.8
Grip force (kg)	24.5 (7.3)	38.9 (9.7)
Self-reported muscle stiffness (%)	23.3	12.8
Smoking (%)	28	37
Alcohol consumption (time/week)	2.0 (1.1)	2.6 (1.2)
Diabetes (%)	6.7	8.6
MMSE (0–30)	28.5 (1.3)	28.3 (1.3)
TMT B-A (s)	50.0 (32.4)	51.6 (32.8)
Reported lifetime depression (%)	29.6	17.4
BDI-I (0–63)	7.2 (6.9)	5.0 (5.2)

Values are presented in mean and (standard deviation). BDI-I, Beck’s depression inventory I; BMI, Body mass index; ISCED, international standard classification of education; MMSE, mini-mental state examination; TMT B-A, trail making test (part B- part A); MDS-UPDRSIII: The Movement Disorder Society-Sponsored Revision of the Unified Parkinson’s Disease Rating Scale, part III.

### Gait assessment, data extraction, and analysis of IMU data

Participants performed 20-m walks in a straight, well-lit, 2-m-wide unobstructed hallway during all four visits. They were asked to walk at a self-selected speed after being equipped with either the Dynaport sensor (McRoberts B.V., The Hague, The Netherlands, in visits 1 and 2) or the Mobility Lab sensor system (APDM Inc., Portland, or, in visits 3 and 4). Raw accelerometer data were extracted from the lower back IMU and analyzed using a validated algorithm (Del Din et al., 2016). We excluded the first three and last three steps to avoid the acceleration and deceleration effects expected during the beginning and end of the walk (Lindemann et al., 2008).

The following parameters of gait (Lord et al., 2013) were analyzed: gait speed (m/s), step length (%/stature) (step length initially calculated using the formula: $\left[Steplength=2\sqrt{2Ih-h^{2}}\right]$ where *h* is the change vertical position of the center of mass, *I* is the pendulum length (sensor height from ground; Del Din et al., 2016), step time (s), stride time [(s; calculated from IC (*i* + 2) − IC(*i*), *i* stands for the step sequence; Del Din et al., 2016)], swing time (s), stance time (s), gait velocity variability (m/s), step length variability (m), step time variability (s), stride time variability(s), swing time variability (s), stance time variability (s), step length asymmetry (m), swing time asymmetry (s), and stance time asymmetry (s). The variability parameters were calculated from the standard deviation of each original parameter (e.g., length, time), and asymmetry was calculated as Average_left_ − Average_right_ of the original parameter (Del Din et al., 2016).

### Statistics

We used IBM SPSS Statistics (Version 24.0, Armonk, NY, USA) for statistical analyses. Data are presented for the overall cohort and for different age groups, always separated by sex. Similar to previous studies (Oberg et al., 1993; Auvinet et al., 2002), we divided the overall cohort into three age groups: 50–59, 60–69, and 70+ years. Significance was set at *p* < 0.05. Exact values are provided in the respective tables. The analysis covered the following two parts.

### Cross-sectional analysis considering age and sex

Gait parameters from baseline data (first visit of each participant) were compared between women and men using a student *t*-test. A simple linear regression model was used to test the effects of age on each gait parameter. The confidence interval was set at 95%.

### Longitudinal analysis

For the 6-year longitudinal analysis, a generalized estimating equation (GEE) model was performed using identity link functions with normal distributions and an exchangeable working correlation structure. All gait parameters underwent a longitudinal analysis using the GEE model.

Differences in longitudinal changes of gait parameters between sex (when stratified for age groups) and age groups (when stratified for sex) were tested based on Time*Sex and Time*Age group interaction effects. GEE models with the subject variable (participant ID), the within-subject variable *Time* (visit number, centered variable), the covariate *Group* (sex: women-0.5; men 0.5; or age group: pairwise group comparisons), and the interaction term (Time*Group) were calculated. Group effects are related to the group difference at *Time*_(centered)_ = 0, i.e., the mean longitudinal observation period. For each gait parameter, annual changes were calculated based on the GEE regression coefficient of *Time* in women, men, and the different age groups, and related to their value of the gait parameter at baseline. Participants who dropped out after two or three visits and those who only joined the study from the second or third visit were still included in the analysis with their respective first visit as their baseline. Only baseline age (at the first visit of each participant) was considered, and participants remained within that age group, and did not change group assignments during aging.

## Results

### Cross-sectional analysis considering sex and age

Detailed information about gait parameters, separated by sex is presented in Table 2. In the overall cohort, six gait parameters were significantly different between men and women. Men had faster gait speed, larger step length, and step duration as well as longer stride, swing and stance times, than women.

**Figure 1:**
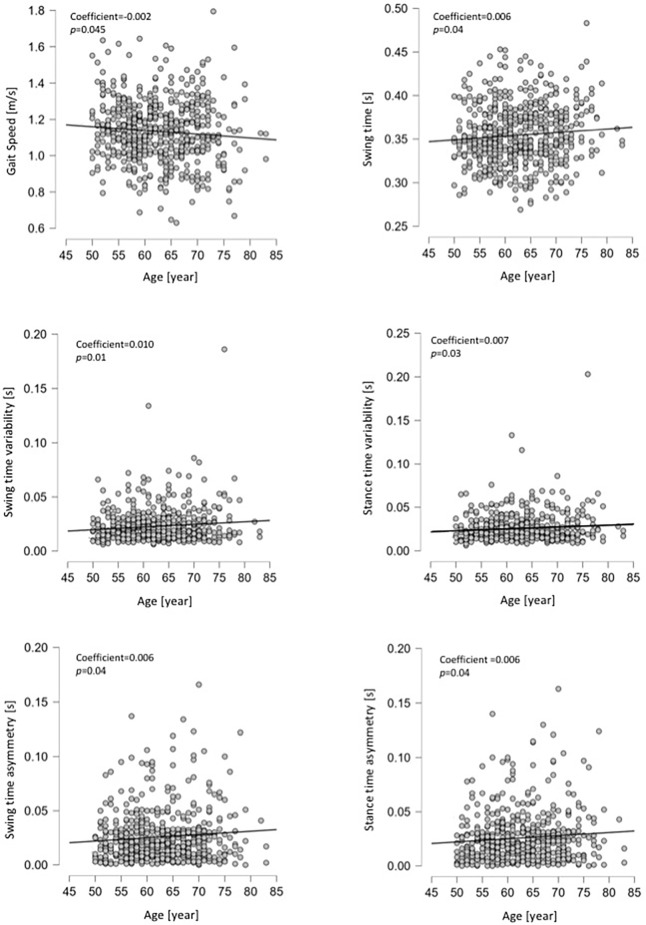
Scatter plot with a fitted line of the linear regression in six gait parameters (in women) were age has significant predictive values.

The linear regression analysis of age as a predictor for the 14 gait parameters in both sexes showed significant p-values in gait speed (slower with increasing age), swing time (longer), and swing and stance time variability and asymmetry (all increasing with age), only in women. Linear regressions did not predict parameter change with age in men. Detailed results of the regression models are presented in Table 3 and Figure 1.

**Table 3:** Cross-sectional analysis of gait parameters separated by sex.

**Parameter**	**Entire cohort (*n* = 1,161)**		***p*-value (*t*-test)**	**Women**			**Men**		
	**Women (*n* = 589)**	**Men (*n* = 572)**		**Coefficient**	**Adjusted R^2^**	***p*-value**	**Coefficient**	**Adjusted R^2^**	***p*-value**
Gait speed (m/s)	1.134 (0.17)	1.155 (0.16)	**0.03**	-0.002	0.005	**0.045**	-0.002	0.004	0.07
Step length (%/stature)	32.26 (3.40)	34.16 (3.43)	**<0.001**	-0.001	0.004	0.08	-0.001	0.004	0.08
Step time (s)	0.51 (0.04)	0.52 (0.05)	**<0.001**	0.0004	0.002	0.14	0.0003	0.001	0.25
Stride time (s)	1.01 (0.08)	1.03 (0.09)	**<0.001**	0.001	0.001	0.19	0.001	0.001	0.25
Swing time (s)	0.35 (0.03)	0.36 (0.04)	**<0.0001**	0.0004	0.006	**0.04**	0.0003	0.001	0.19
Stance time (s)	0.66 (0.06)	0.67 (0.06)	**0.003**	0.0002	-0.001	0.48	0.0002	-0.0001	0.40
Gait speed var(s)	0.089 (0.040)	0.087 (0.037)	0.44	0.0001	-0.002	0.07	0.0002	0.001	0.28
Step length var(s)	0.035 (0.016)	0.034 (0.013)	0.06	0.0001	-0.00001	0.37	0.0001	-0.00001	0.40
Step time var (s)	0.028 (0.02)	0.029 (0.02)	0.30	0.0002	0.004	0.07	0.113	-0.00001	0.32
Stride time var (s)	0.025 (0.033)	0.024 (0.014)	0.56	0.0001	-0.002	0.70	0.128	0.002	0.13
Swing time var (s)	0.023 (0.015)	0.023 (0.014)	0.45	0.0002	0.010	**0.01**	0.0001	-0.001	0.45
Stance time var (s)	0.025 (0.016)	0.026 (0.014)	0.81	0.0002	0.007	**0.03**	0.0001	-0.00001	0.36
Step length asy (m)	0.026 (0.022)	0.026 (0.023)	0.94	0.0001	-0.001	0.60	0.257	0.004	0.07
Swing time asy (s)	0.026 (0.024)	0.027 (0.027)	0.27	0.0003	0.006	**0.04**	0.00002	-0.002	0.90
Stance time asy (s)	0.026 (0.023)	0.027 (0.027)	0.38	0.0003	0.006	**0.04**	0.000002	-0.002	0.99

Student’s *t*-test analyses of gait parameters in both sexes and linear regression with age as a predictor of changes in gait parameters. Significant p-values are presented in bold.

### Longitudinal analysis

**Figure 2:**
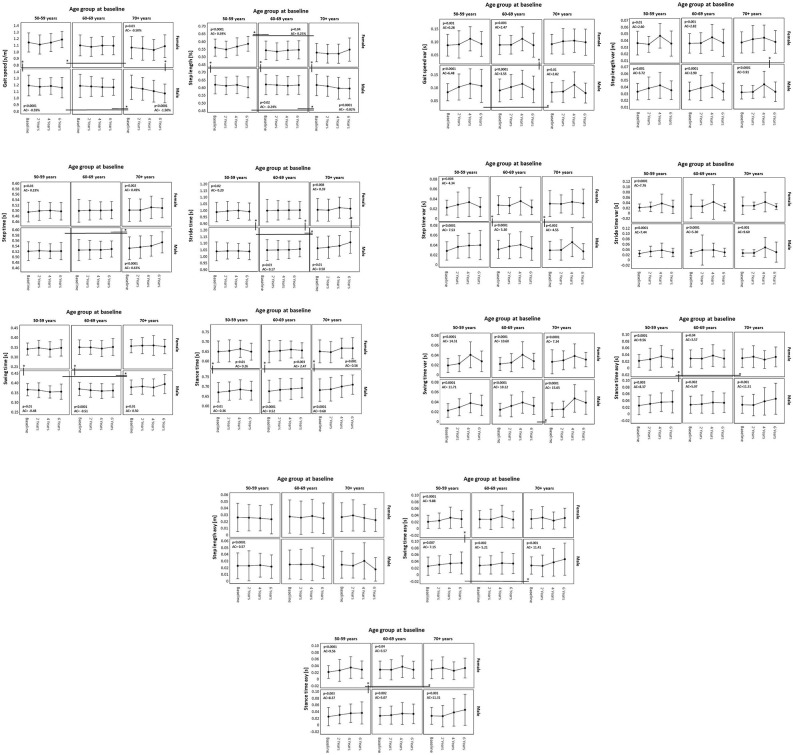
Change in gait parameters (means and standard deviations) over 6 years in women and men per age group, extracted from a generalized estimating equation model. AC, annual change; *significant difference (*p* < 0.05).

The GEE results reflect the changes over time in the gait parameters included in this analysis, in each sex and age group (in decades) over the period of 6 years including the annual change in each parameter. Details are provided in Table 4 and Figure 2. Overall, the following patterns could be observed: Means of temporal and spatial parameters showed plausible changes over the individual visits; in men, a continuous deterioration of these parameters could be detected, especially in the 70+ group. In women, there was no definite evidence of an age-associated deterioration of gait parameters detectable. Variability and asymmetry parameters showed a less clearly age-associated pattern of change, with temporal parameters showing clearer age associations than spatial parameters, especially in men.

**Table 4:** Mean performance and annual change over 6 years observation period of women and men separated by age groups.

	**Women**														
	**50–59 (*N* = 217)**					**60–69 (*N* = 263)**					**70+ (*N* = 109)**				
	**Mean (SD)**	**Reg. Coeff. B**	**Std. error**	**AC%**	***p***	**Mean (SD)**	**Reg. Co. B**	**Std. error**	**AC%**	***p***	**Mean (SD)**	**Reg. Coeff. B**	**Std. error**	**AC%**	***p***
Gaitspeed (m/s)	1.14 (0.14)	0.01	0.004	0.24	0.14	1.10 (0.16)	−0.004	0.004	−0.20	0.28	1.06 (0.17)	−0.01	0.01	−0.50	**0.03**
Steplength (%/stature)	33.7 (3.10)	0.04	0.08	0.59	**<0.0001**	33.1 (3.53)	0.17	0.08	0.25	**0.04**	32.1 (3.33)	0.08	0.12	0.52	0.52
Step time (s)	0.49 (0.04)	0.002	0.01	0.19	**0.03**	0.50 (0.04)	0.002	0.001	0.16	0.10	0.50 (0.04)	0.01	0.002	0.49	**0.002**
Stride time (s)	0.99 (0.07)	0.004	0.002	0.20	**0.02**	1.00 (0.09)	0.004	0.002	0.19	0.056	1.01 (0.09)	0.008	0.002	0.39	**0.008**
Swing time (s)	0.34 (0.3)	0.001	0.001	0.10	0.54	0.35 (0.04)	0.0001	0.001	−0.03	0.85	0.35 (0.03)	0.002	0.002	0.25	0.32
Stance time (s)	0.65 (0.54)	0.003	0.001	0.26	**0.01**	0.65 (0.06)	0.004	0.001	0.31	**0.003**	0.65 (0.06)	0.007	0.002	0.56	**0.001**
Gaitspeedvar (s)	0.09 (0.04)	0.005	0.002	0.28	**0.001**	0.09 (0.04)	0.004	0.002	2.47	**0.003**	0.09 (0.04)	0.005	0.003	2.80	0.07
Steplengthvar (s)	0.02 (0.02)	0.002	0.001	2.60	**0.01**	0.03 (0.02)	0.002	0.001	2.82	**0.001**	0.04 (0.02)	0.002	0.001	2.82	0.051
Step time var (s)	0.02 (0.02)	0.002	0.001	4.34	**0.004**	0.03 (0.02)	0.001	0.001	1.38	0.27	0.03 (0.03)	0.001	0.002	1.69	0.49
Stride time var (s)	0.02 (0.01)	0.003	0.001	7.76	**<0.0001**	0.03 (0.04)	0.002	0.002	4.59	0.11	0.03 (0.03)	0.003	0.002	4.50	0.11
Swing time var (s)	0.02 (0.01)	0.006	0.001	14.31	**<0.0001**	0.02 (0.01)	0.005	0.001	10.6	**<0.0001**	0.03 (0.02)	0.004	0.001	7.34	**<0.0001**
Stance time var (s)	0.02 (0.01)	0.005	0.001	11.21	**<0.0001**	0.03 (0.01)	0.004	0.001	7.89	**<0.0001**	0.03 (0.02)	0.004	0.001	6.92	**0.003**
Steplengthasy (m)	0.03 (0.02)	−0.001	0.001	-1.33	0.36	0.03 (0.03)	<0.0001	0.001	−0.21	0.88	0.03 (0.02)	−0.001	0.001	−1.04	0.64
Swing time asy (s)	0.02 (0.02)	0.004	0.001	9.88	**<0.0001**	0.03 (0.03)	0.002	0.001	3.06	0.7	0.03 (0.03)	<0.0001	0.002	0.43	0.87
Stance time asy (s)	0.02 (0.02)	0.004	0.001	9.56	**<0.0001**	0.03 (0.03)	0.002	0.001	3.57	**0.04**	0.03 (0.03)	<0.0001	0.002	0.79	0.76
	**Men**														
	**50–59 (*N* = 135)**					**60–69 (*N* = 301)**					**70+ (*N* = 136)**				
	**Mean (SD)**	**Reg. Coeff. B**	**Std. error**	**AC%**	***p***	**Mean (SD)**	**Reg. Co. B**	**Std. error**	**AC%**	***p***	**Mean (SD)**	**Reg. Coeff. B**	**Std. error**	**AC%**	***p***
Gaitspeed (m/s)	1.19 (0.15)	−0.01	0.01	-0.28	0.17	1.18 (0.17)	−0.13	0.004	−0.59	**<0.0001**	1.16 (0.18)	−0.04	0.005	−1.50	**<0.0001**
Steplength (%/stature)	34.4 (3.20)	−0.07	0.09	-0.10	0.44	34.8 (3.63)	−0.17	0.07	−0.24	**0.02**	34.6 (3.80)	−0.57	0.12	−0.82	**<0.0001**
Step time (s)	0.52 (0.04)	0.001	0.001	0.08	0.44	0.52 (0.04)	0.001	0.001	0.14	0.09	0.53 (0.04)	0.01	0.001	0.63	**<0.0001**
Stride time (s)	1.04 (0.07)	0.001	0.002	0.07	0.51	1.05 (0.08)	0.004	0.002	0.17	**0.03**	1.06 (0.08)	0.014	0.003	0.65	**<0.0001**
Swing time (s)	0.37 (0.03)	−0.003	0.001	-0.48	**0.01**	0.37 (0.03)	−0.004	0.001	−0.51	**<0.0001**	0.38 (0.04)	0.004	0.002	0.50	**0.01**
Stance time (s)	0.67 (0.05)	0.005	0.002	0.36	**0.01**	0.68 (0.06)	0.007	0.001	0.52	**<0.0001**	0.68 (0.06)	0.009	0.002	0.68	**<0.0001**
Gaitspeedvar (s)	0.08 (0.03)	0.011	0.002	6.48	**<0.0001**	0.08 (0.04)	0.006	0.002	3.55	**<0.0001**	0.08 (0.04)	0.005	0.002	2.82	**0.01**
Steplengthvar (s)	0.03 (0.01)	0.002	0.001	3.72	**0.001**	0.03 (0.01)	0.002	0.001	2.90	**<0.0001**	0.03 (0.01)	0.002	0.001	3.91	**<0.0001**
Step time var (s)	0.03 (0.02)	0.004	0.001	7.53	**<0.0001**	0.03 (0.02)	0.003	0.001	5.30	**<0.0001**	0.03 (0.02)	0.003	0.001	4.55	**0.002**
Stride time var (s)	0.02 (0.01)	0.003	0.001	7.44	**<0.0001**	0.03 (0.01)	0.003	0.001	5.30	**<0.0001**	0.02 (0.01)	0.005	0.001	9.60	**0.001**
Swing time var (s)	0.02 (0.01)	0.005	0.001	11.71	**<0.0001**	0.02 (0.01)	0.005	0.001	10.12	**<0.0001**	0.02 (0.01)	0.007	0.001	15.65	**<0.0001**
Stance time var (s)	0.02 (0.01)	0.005	0.001	10.18	**<0.0001**	0.03 (0.01)	0.004	0.001	8.08	**<0.0001**	0.03 (0.01)	0.007	0.001	12.91	**<0.0001**
Steplengthasy (m)	0.02 (0.02)	0.024	0.001	0.57	**<0.0001**	0.03 (0.02)	<0.0001	0.001	0.17	0.88	0.03 (0.02)	<0.0001	0.001	0.40	0.83
Swing time asy (s)	0.03 (0.03)	0.004	0.001	7.15	**0.007**	0.03 (0.03)	0.003	0.001	5.21	**0.002**	0.03 (0.03)	0.006	0.002	11.41	**0.001**
Stance time asy (s)	0.03 (0.03)	0.004	0.001	8.37	**0.003**	0.03 (0.03)	0.003	0.001	5.07	**0.002**	0.03 (0.03)	0.006	0.002	11.31	**0.001**

Baseline assessment presented with mean (standard deviation). Annual change (AC) in percent calculated with the general estimation equation. Reg. coeff. B, regulated coefficient B; SD, standard deviation; Std error, Standard Error. Significant p-values are presented in bold.

## Discussion

In this study, we present cross-sectional and longitudinal data of gait parameters under a self-selected speed condition using a single IMU on the lower back in a large cohort of older adults. While the data provided here does not in itself define normal and abnormal gait in older adults, it can help future studies by acting as reference data. Differences between our values and from previous datasets data can be explained by different settings, cohorts, and protocols. For example, most of the previous studies adopted shorter distances of walking, used less granular (stopwatch) or more artificial assessment strategies (treadmill), and investigated smaller cohorts (Oberg et al., 1993; Auvinet et al., 2002; Ko et al., 2010; Bohannon and Williams Andrews, 2011).

In so far, this study adds new information to already existing datasets by: (i) investigating a large cohort of women and men during a period when age-associated diseases have a relevant influence on gait, (ii) with modern wearable technology and a validated algorithm (Del Din et al., 2016), (iii) using a paradigm relevant for daily living allowing a reliable gait analysis (Lindemann et al., 2008), and (iv) performing longitudinal follow-ups to sufficiently delineate average annual changes by sex and decades of age.

In agreement with previous studies (Kerrigan et al., 1988b; Lee and Grimson, 2002; Bohannon and Williams Andrews, 2011; Ko et al., 2011; Makihara et al., 2011), our data show significant sex differences in gait speed, step length and time, stride, and swing time (women < men). Our sex-specific gait differences are supported and explained, by results from biomechanics studies showing that men and women have different mechanics when walking (Kerrigan et al., 1988a; Smith et al., 2002). For example, women have more pelvic obliquity range and less center of mass vertical displacement that men (Smith et al., 2002). They also have less knee extension before the initial contact and a greater knee flexion in pre-swing phase than men (Kerrigan et al., 1988a). Thus, our dataset can be used, for example, to develop sex identification classifiers in these age groups (Kerrigan et al., 1988b; Lee and Grimson, 2002). Interestingly, differences were temporal parameters, making the parameters particularly interesting for investigations in this field (Lee and Grimson, 2002). *Vice versa*, studies investigating gait in older cohorts should account for this sex effect on the above-mentioned parameters.

Our cross-sectional analyses with a prediction of age effects suggest that many gait parameters are relatively preserved into old age. This effect may be partly explained by a comparably “healthy” and well-educated cohort (see limitations) that was investigated here. However, there is also existing literature on this topic, showing that about 20% of individuals beyond the age of 88 years do not have any gait deficit (Lagaay et al., 1992). In our opinion, these data confirm the authors’ hypothesis that the term “age-related gait deficits” should be used very carefully, and that it is very likely that these gait changes are mostly caused by (age-related) diseases. Basically, our results suggest that the paradigm presented here is plausible and can be used for the detection of such gait changes.

Our longitudinal analyses, separated by sex and divided into decades of age, showed that especially the means of temporal and spatial parameters changed continuously over the individual visits. One example was gait speed. Self-selected gait speed is a robust indicator of vitality and its decline is often a consequence of disturbances in at least one of our body systems, including the brain (Abellan Van Kan et al., 2009; Studenski et al., 2011). We found that in men, a significant continuous annual change in gait speed started from the 7th decade and increased in the 8th. Women showed a trend toward continuous and reliable reduction of gait speed about 10 years later. Our findings are comparable with previous studies, which showed a constant reduction in gait speed in older adults, especially from the 7th decade on (Abellan Van Kan et al., 2009; Studenski et al., 2011). It is important to note that the association between some gait parameters means that the decline in gait speed can be a result of the decline in other closely related parameters (e.g., step time or step length), and also *vice versa*. The combination of increased step time and reduced step length leads necessarily to lowered gait speed. Therefore, men are obviously more (or at an earlier point in time) prone than women to a continuous reduction of gait speed during aging. Our results confirm results from previous studies investigating gait speed in both sexes (Bohannon and Williams Andrews, 2011).

Step length showed a trend of decline in women from the 7th decade on, which reached significance in the 8th decade. Men in the 8th decade showed a significant and continuous decline in step length in the 6-year follow-up, making step length especially in older men a particularly promising and relatively fine-granular measure for age-related changes. Possible causes of step length reduction in older adults include, but are not limited to, skeleton muscular pathologies, fear of falling, and neurodegenerative diseases (Judge et al., 1996a, b). The male dominance of clinical and prodromal phases of common diseases, such as Parkinson’s disease, stroke, and heart diseases, may further explain these findings (Bloem et al., 2000).

This study has some limitations. The TREND cohort has a relatively large proportion of participants who are highly educated [47% completed a tertiary level of education, which is a higher percentage than in the European population (32%) and in overall Germany (28%)]^2^. Still, we feel that our study can serve as a reference dataset for future studies at least in highly developed countries given the trends of improved lifestyle and health status during recent decades, and their influence on age-related function and disease. Furthermore, this study used a laboratory setting. It would be interesting for future research to explore gait in a more natural environment. Nonetheless, the advantage of the method is the high degree of standardization and, consequently, high validity and reliability.

As in all longitudinal observational studies, also this study had some attrition over consecutive visits, and we cannot exclude that those who did not remain in the study may have become ill, less mobile, etc., and, therefore, this may have had an influence on our results. However, we argue that we had exceptionally good retention rates over the visits, compared to other studies in the field. It would also be interesting to compare the performance of participants who remained in the study for over 6 years to those who dropped out. It is not unlikely that participants who remained in the study for over 6 years are “over performers” who do not accurately represent this age group.

A further limitation was the lack of specific information about pain, which may have a relevant influence on gait parameters (Kirmizi et al., 2019). Nonetheless, we believe that our large cohort can represent the older adults in its community. For validation purposes, a future study with repeated assessments would be beneficial to assess the test/retest reliability of IMU-based gait parameters. This seems especially relevant for variability and asymmetry parameters. We expect a deterioration with age in some aspects of these parameters as well (Hirono et al., 2021; Kwek and Williams, 2021), and indeed found this for the temporal parameters (for men). For the spatial parameters, however, we could not find any clear age associations, which might also be due to the potentially low reliability of these parameters when measured with the paradigm presented here (Almarwani et al., 2016). It could also theoretically be that the use of different IMUs influences the variability of the data, particularly in spatial parameters. However, both devices used in this study are certified reliable systems and differences in raw data between such systems are negligible.

In summary, this study presents a large dataset of gait parameters collected cross-sectionally and longitudinally in an older cohort with a lower back-worn IMU. Results indicate that men are likely to show age-associated gait changes earlier than women, and that temporal gait parameters in particular show plausible and continuous changes over observation periods of 2 years. This dataset and the results may provide important guidance for promising gait outcome parameters for future observational and therapeutic studies planning assessment with a lower back IMU.

## Data Availability Statement

The raw data supporting the conclusions of this article will be made available by the authors, without undue reservation.

## Ethics Statement

The studies involving human participants were reviewed and approved and the study was approved by the ethics committee of the medical faculty of Eberhard Karls University of Tübingen and the University Hospitals of Tübingen (Nr. 90/2009BO2). The patients/participants provided their written informed consent to participate in this study.

## Authors Contributions

ME: project conceptualization, data acquisition, statistical design and analysis, first draft writing. SD: project conceptualization, data analysis, critical revision of the manuscript for important intellectual content. MH: data acquisition, project conceptualization, manuscript revision. EW: statistical analysis, manuscript critical revision. US: study design and organization, data acquisition, manuscript revision. TS-H: statistical design, manuscript revision. LB: statistical analysis. SH: statistical analysis, first draft writing, manuscript revision. KB, FM, and DB: study design and organization, manuscript revision. CS and CH: statistical analysis, manuscript revision. LR: project conceptualization, interpretation of data, manuscript revision. WM: study design and organization, project conceptualization, statistical design, manuscript writing and revision. All authors contributed to the article and approved the submitted version.

## Funding

We would also like to acknowledge the support by the Deutsche Forschungsgemeinschaft and Open Access Publishing Fund of Tübingen University, Tübingen, Germany. SD is supported by the Newcastle Biomedical Research Centre (BRC) based at Newcastle upon Tyne and Newcastle University. The work was also supported by the NIHR/Wellcome Trust Clinical Research Facility (CRF) infrastructure at Newcastle upon Tyne Hospitals NHS Foundation Trust. All opinions are those of the authors and not the funders. EW is supported by Keep Control from the European Union’s Horizon 2020 research and innovation programme under the Marie Skłodowska-Curie grant agreement No. 721577. All opinions are those of the authors and not the funders.

## Footnotes

^1^ https://www.trend-studie.de

^2^ https://gpseducation.oecd.org/
